# Control of proline utilization by the Lrp-like regulator PutR in *Caulobacter crescentus*

**DOI:** 10.1038/s41598-018-32660-3

**Published:** 2018-10-02

**Authors:** Annabelle Mouammine, Katharina Eich, Antonio Frandi, Justine Collier

**Affiliations:** Department of Fundamental Microbiology, Faculty of Biology and Medicine, University of Lausanne, Quartier UNIL/Sorge, Lausanne, CH 1015 Switzerland

## Abstract

Cellular metabolism recently emerged as a central player modulating the bacterial cell cycle. The *Alphaproteobacterium Caulobacter crescentus* appears as one of the best models to study these connections, but its metabolism is still poorly characterized. Considering that it lives in oligotrophic environments, its capacity to use amino-acids is often critical for its growth. Here, we characterized the *C. crescentus* PutA bi-functional enzyme and showed that it is required for the utilization of proline as a carbon source. We also found that *putA* transcription and proline utilization by PutA are strictly dependent on the Lrp-like PutR activator. The activation of *putA* by PutR needs proline, which most likely acts as an effector molecule for PutR. Surprisingly, we also observed that an over-production of PutR leads to cell elongation in liquid medium containing proline, while it inhibits colony formation even in the absence of proline on solid medium. These cell division and growth defects were equally pronounced in a Δ*putA* mutant background, indicating that PutR can play other roles beyond the control of proline catabolism. Altogether, these findings suggest that PutR might connect central metabolism with cell cycle processes.

## Introduction

The proline amino-acid is not only indispensable for protein synthesis, but also often for osmoprotection or heavy metal tolerance^1,2^. In addition, proline can be used as a source of nutrients by bacteria leaving in poor nutrient conditions or in proline-rich host microenvironments^3^. The first step of the proline utilization pathway is the conversion of proline into glutamate, to feed the tricarboxylic acid (TCA) or the nitrogen cycles. This conversion requires the sequential action of a proline dehydrogenase (PRODH) and of a Δ^1^-pyrroline-5-carboxylate dehydrogenase (P5CDH). The first of these two oxidation reactions produces reactive oxygen species as frequent by-products^4–7^. In most but not all bacteria, both activities are carried by a single bi-functional enzyme called PutA^3^. This reaction is not only necessary for proline utilization, but also for a diversity of biological processes by bacteria such as infection, symbiosis and redox homeostasis^3,7–9^.

Considering the multiple roles of PutA inside cells, its activity must be tightly controlled. While the enzymes carrying PRODH and P5CDH domains are highly conserved, the control of their levels or activities is much more divergent. It is especially obvious in bacteria, where *putA* transcription is highly controlled by one or more transcription factors in response to nutrient availability^10,11^. In some bacteria, such as *Escherichia coli* or *Salmonella thyphimurium*, PutA has a DNA binding domain and can repress *putA* transcription^12,13^, while other bacteria, such as *Agrobacterium tumefaciens* or *Brucella abortus* encode an independent regulator named PutR to control *putA* transcription^9,14^. *putA* is sometimes regulated by PutA and PutR such as in *Rhodobacter capsulatus*^15^, illustrating the diversity of pathways regulating proline utilization in bacteria. The PutR regulator belongs to the Lrp/AsnC family of transcriptional regulators that are the most often responding to effector molecules^16,17^. This family includes not only specific regulators, but also global regulators controlling large regulons. Several members of this family respond to amino-acids, such as leucine for the best characterized leucine-responsive protein (Lrp)^16^. As for PutR, it has been shown to respond to proline in bacteria where it has been investigated^11,14,15^. Proline binding to PutR most likely induces a conformational change necessary for its binding to the *putA* promoter. It nevertheless remains unclear whether PutR proteins are always specific regulators of *putA*. Interestingly, one study indicated the PutR protein of *Agrobacterium tumefaciens* has the capacity to condense DNA into larger nucleoprotein complexes^18^, suggesting that its mechanism of action is original in *Alphaproteobacteria*. It may control other genes or affect chromosome conformation as seen for some other Lrp-like factors^16^.

*Caulobacter crescentus* is a well-characterized *Alphaproteobacterium*, as it serves as an excellent model system to study the bacterial cell cycle and the biology of the bacterial chromosome^19,20^. Interestingly, several studies recently uncovered interesting connections between metabolic pathways and cell cycle progression in this bacterium^21–26^. This might be particularly important for oligotrophic bacteria like *C. crescentus*, which thrives in aquatic environments such as lakes, rivers or oceans. In such environments, nutrients are rare and very diverse. Then, *C. crescentus* must be able to use a variety of different carbon sources and to modulate its cell cycle in response to nutrient availability as a strategy to survive in its natural environment. The central metabolism of *C. crescentus* and its control is however underexplored^27^. Still, it is known that this bacterium can use many sugars and amino-acids, including proline, as carbon sources^28–31^.

In this study, we characterized the proline utilization pathway of *C. crescentus* and its control in response to proline availability. In addition, we uncovered a potential connection between proline catabolism and cell division through the Lrp-like PutR regulator.

## Results

### *Caulobacter crescentus* needs PutA for proline utilization

**Figure 1:**
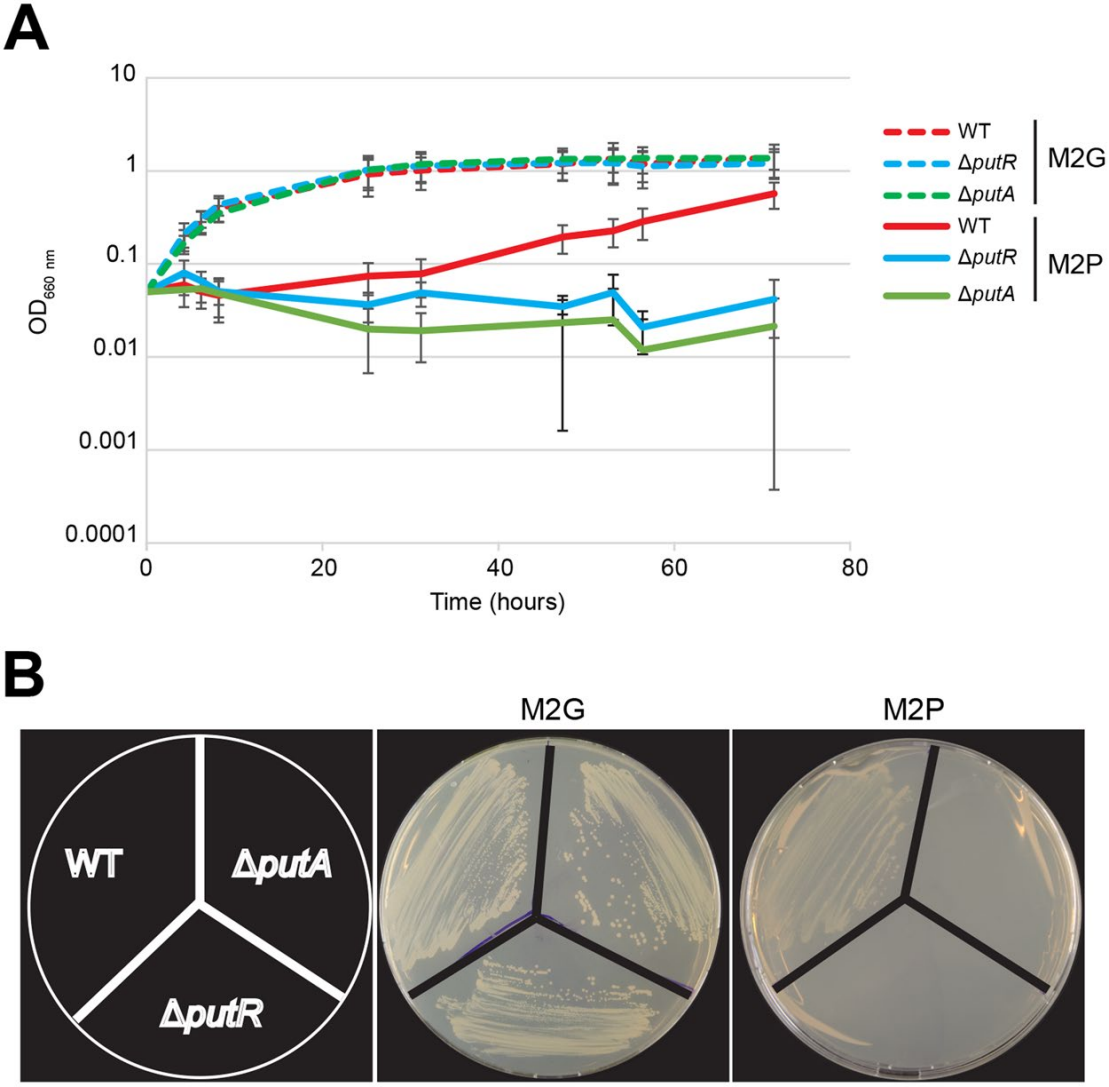
PutA and PutR are required for proline utilization as a carbon source. (**A**) Growth of WT (NA1000), Δ*putA* (JC1695) and Δ*putR* (JC1040) strains in liquid M2 minimal media with 0.2% glucose (M2G, dashed lines) or 10 mM proline (M2P, solids lines) as carbon sources. Each culture was cultivated over-night in M2G medium and re-diluted into M2G or M2P media at time 0. Growth was tracked by measuring the OD_660nm_ for ~70 hours. Each curve represents the mean of six independent experiments. Error bars correspond to standard deviations. (**B**) Growth of WT, Δ*putA* and Δ*putR* colonies on solid M2 minimal medium with 0.2% glucose (M2G) or 10 mM proline (M2P) as carbon sources. Representative plates (from three independent experiments) incubated for 6 days are shown here.

Most bacteria use a bi-functional PutA enzyme to catabolize the conversion of proline into glutamate, the first step in proline utilization^3^. In order to study how *C. crescentus* uses proline as a source of nutrients, we searched for a *putA* homolog in its genome and found the CCNA_00846 protein that shares ~47% of identity with bi-functional PutA proteins from other *Alphaproteobacteria*^9,14^. It encodes a protein carrying two domains: one with similarities with the PRODH domain and the other one with similarities with the P5CDH domain, as expected for a bi-functional enzyme^3^. We constructed a complete Δ*putA* deletion strain and found that the growth rate (Supplementary Fig. S1) and the morphology (Supplementary Fig. S2) of mutant cells cultivated in complex peptone-yeast extract (PYE) medium (in which amino-acids serve as the primary carbon source) were unaffected, compared to isogenic wild-type (WT) cells. We then tested its capacity to grow in minimal media with proline (M2P) or glucose (M2G) as the sole carbon source. As expected, the Δ*putA* and its isogenic wild-type (WT) strain could both grow at a similar rate in liquid (Fig. 1A) or solid (Fig. 1B) M2G. We also observed that the WT strain could grow on M2P, but at a lower rate than on M2G, showing that glucose is a better carbon source than proline for *C. crescentus*. As for the Δ*putA* strain, we could not detect any growth in liquid (Fig. 1A) or on solid (Fig. 1B) M2P media, showing that PutA is required for proline utilization by *C. crescentus*. This result strongly suggests that *C. crescentus* PutA can convert proline into glutamate as previously shown for PutA homologs from other *Alphaproteobacteria*^9,11,14,15^.

### PutR is critical for *putA* transcription and proline utilization

**Figure 2:**
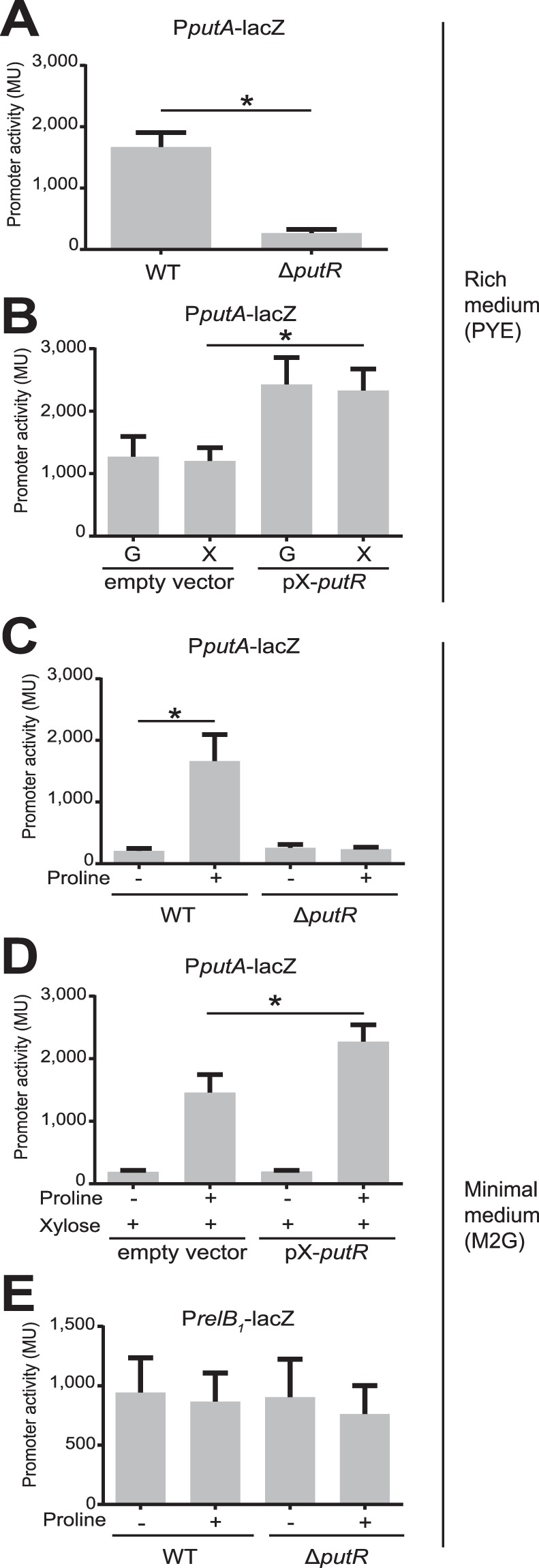
PutR is a proline-dependent activator of the *putA* but not of the *relB*_1_ promoters. Promoter activities in Miller Units (MU) were measured by β-galactosidase assays from cells cultivated in exponential phase and carrying the p*lacZ*290-*putA*P (P*putA*-l*acZ*) or p*lacZ*290-*relB*_1_P (P*relB*_1_-l*acZ*) plasmids. Error bars correspond to standard deviations from minimum six independent experiments. Statistical analysis were performed using student t-tests. Stars (*) highlight significant (p < 0.0005) but also relevant differences between two strains. (**A**) Activity of the *putA* promoter in WT (NA1000) or Δ*putR* (JC1040) strains cultivated in PYE rich medium. (**B**) Activity of the *putA* promoter in a WT strain carrying pBXMCS6 (empty vector) or pX-*putR*. Cells were cultivated over-night and re-diluted into PYE + 0.2% glucose (PYEG). Once cells reached exponential phase again, 0.3% xylose was added into half of the culture (PYEGX) to induce the expression of *putR* from pX-*putR*. β-galactosidase assays were done 90 minutes after. (**C**) Activity of the *putA* promoter in WT or Δ*putR* strains cultivated in M2 minimal medium containing 0.2% glucose (M2G) supplemented or not with 10 mM proline. (**D**) Activity of the *putA* promoter in a WT strain carrying pBXMCS6 (empty vector) or pX-*putR*. Cells were cultivated over-night in M2G and re-diluted into M2G supplemented or not with proline. Once cells reached exponential phase again, 0.3% xylose was added into half of the culture to induce the expression of *putR* from pX-*putR*. β-glactosidase assays were done 180 minutes after. **(E**) Activity of the *relB*_1_ promoter in WT or Δ*putR* strains cultivated in M2 minimal medium containing 0.2% glucose (M2G) supplemented or not with 10 mM proline.

*C. crescentus* must control PutA levels or activity to make sure that it does not catabolize proline when it grows in a medium that does not contain proline, in order to maintain intracellular levels of proline that are sufficient for efficient protein synthesis. Interestingly, a gene (*CCNA_00847*) encoding a protein sharing ~40% of identity with PutR from other *Alphaproteobacteria*^9,14^ lies next to the *putA* gene but in an opposite orientation, suggesting that it may encode a regulator of *putA* transcription. To test if PutR regulates *putA* transcription, we cloned the *putA* promoter region upstream of the *lacZ* gene and introduced this P*putA-lacZ* reporter into a WT, a Δ*putR* deletion strain and a strain over-expressing *putR*. We then used these strains to perform β-galactosidase assays to evaluate the impact of PutR on *putA* transcription in complex PYE medium. We observed that the *putA* promoter was ~7-fold less active in Δ*putR* cells than in WT cells (Fig. 2A), reaching very basal levels that are most likely not sufficient for proline utilization. Indeed, we also observed that Δ*putR* cells could not grow in M2P medium containing proline as the only carbon source, while they could grow as WT cells in M2G (Fig. 1) or PYE media (Supplementary Fig. S1). In addition, the activity of the *putA* promoter was induced ~2-fold upon *putR*-overexpression when cells were cultivated in rich medium for 90 minutes (Fig. 2B). All together, these results show that PutR is a strong activator of *putA* transcription and that PutR is thus required for proline utilization by *C. crescentus*.

### PutR is a proline-responsive activator of *putA*

PutR belongs to the Lrp/AsnC family of transcription factors that often use effector molecules, such as amino-acids, to modulate their activity^16^. Considering that PutR regulates the proline utilization enzyme PutA, we tested if proline may affect the capacity of PutR to activate *putA* transcription. We grew the WT, Δ*putR* and *putR*-over-expression strains carrying the P*putA-lacZ* reporter in M2G media supplemented or not with proline and performed β-galactosidase assays. In the WT strain, the activity of the *putA* promoter was ~8-fold higher when proline was added into the medium, showing that proline induces *putA* transcription (Fig. 2C). Consistent with the proposal that PutR senses proline levels, we observed that proline could not induce the *putA* promoter in the absence of PutR. Interestingly, proline activated the *putA* promoter even more efficiently (~11-fold) when *putR* was over-expressed (Fig. 2D). Overall, these observations show that proline is required for the activation of *putA* transcription by PutR.

### The overproduction of PutR in proline-containing medium leads to cell elongation in a PutA-independent manner

**Figure 3:**
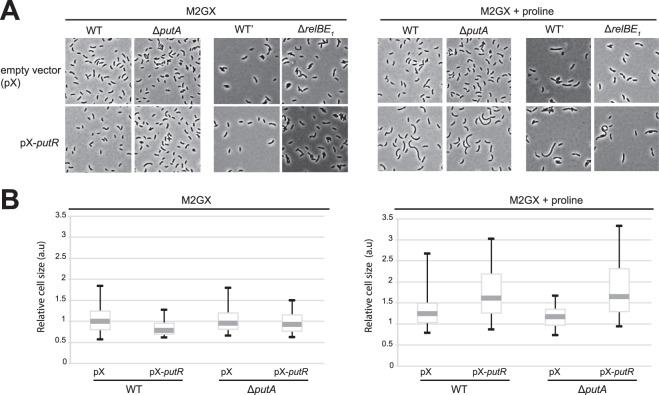
Excess of PutR leads to cell division defects in a proline-dependent manner. NA1000 (WT) and isogenic Δ*putA* cells, or CB15 (WT’) and isogenic Δ*relBE*_1_ cells, carrying the pBXMCS6 (pX; empty vector) or the pX-*putR* plasmid were cultivated in M2G and diluted the next morning into M2G supplemented or not with proline. Once cultures reached an OD_660nm_ of ~0.25, 0.3% xylose was added to induce the expression of *putR* from pX-*putR* over-night. (**A**) Cells were then imaged by phase-contrast microscopy and representative images from three independent experiments are shown in this figure. (**B**) Cell size distributions of WT and Δ*putA* cells carrying pX or pX-*putR* were compared. The bold grey line indicates the median medial axis length of cells. The limits of the box are the first and third quartiles. The whiskers are the 5% and 95% quantiles. The values were normalized so that the median length of the WT cells carrying pX equals 1 arbitrary unit (a.u.) to facilitate comparisons.

Surprisingly, we observed that the overexpression of *putR* under the control of the *xylX* promoter on the medium-copy number vector pBXMCS6 leads to cell morphology defects. Wild-type cells over-expressing *putR* appeared significantly elongated compared to control cells carrying the empty vector, but only when cultivated in the presence of proline in minimal medium (Fig. 3) or in complex medium (Supplementary Fig. S3). This observation suggests that PutR inhibits cell division in liquid cultures. Since this phenotype is dependent on the presence of proline, we hypothesized that it might be connected with proline catabolism due to excessive transcription of *putA* when PutR is over-produced. Indeed, previous studies indicated that proline catabolism produces hydrogen peroxides, which can influence redox homeostasis in certain bacterial species^5,6^. Considering that oxidative stress may lead to DNA or protein damage and that such damage can lead to cell cycle arrests^32,33^, it could have been an explanation for the phenotype of cells over-expressing *putR*. To test this hypothesis, we looked at the effect of PutR overproduction in Δ*putA* cells that cannot catabolize proline. We observed that the cell division defect was similar in wild-type and Δ*putA* cells (Fig. 3), indicating that PutR can inhibit cell division independently of its function as a transcriptional activator of *putA*.

### The overproduction of PutR inhibits colony formation in a PutA-independent manner

**Figure 4:**
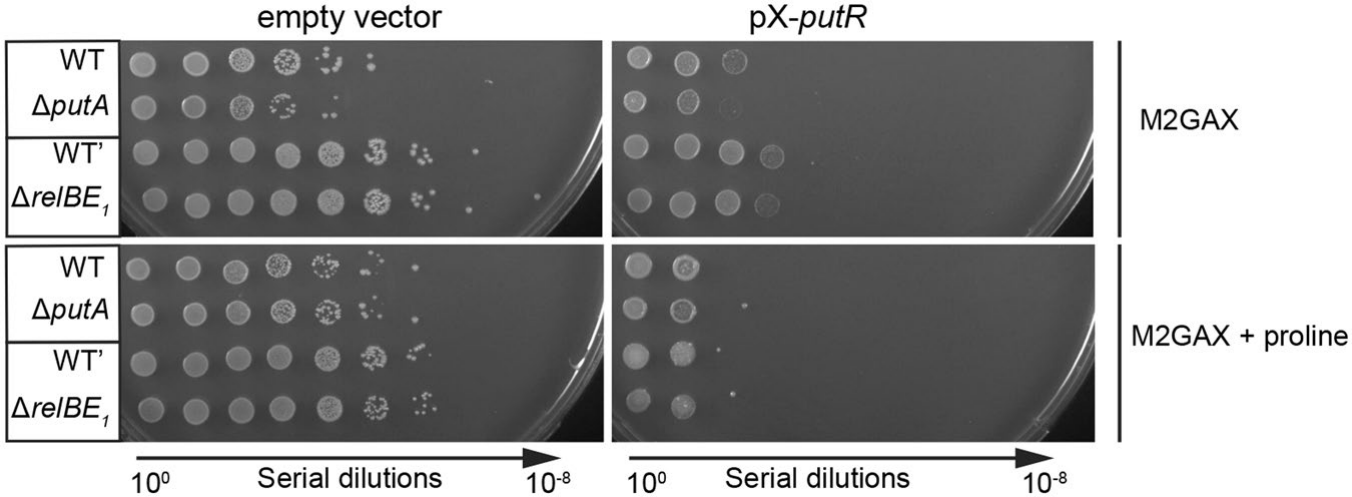
Excess of PutR inhibits colony formation and growth in a proline-independent manner. NA1000 (WT) and isogenic Δ*putA* cells, or CB15 (WT’) and isogenic Δr*elBE*_1_ cells, carrying the pBXMCS6 (empty vector) or the pX-*putR* plasmid were cultivated in M2G and diluted the next morning into M2G supplemented or not with 10 mM proline. Once cultures reached stationary phase, 10-fold serial dilutions in M2G medium were prepared and 5 μL of each dilution was spotted onto M2GA plates containing 0.3% xylose (M2GAX) to induce the expression of *putR* from pX-*putR* over-night. Proline was also added into plates when indicated. Representative images from three independent experiments are shown in this figure.

In addition to the observed cell division defect, we noticed that wild-type cells over-expressing *putR* while cultivated on plates could rarely form colonies (Fig. 4). When growth could nevertheless be detected, colonies appeared as much smaller than colonies of cells carrying the empty vector. Interestingly, this additional phenotype associated with the over-production of PutR was neither dependent on the presence of proline in the medium, nor on the capacity of cells to catabolize proline using PutA. Thus, it appeared as independent of the effect of PutR on *putA* regulation or on cell division.

### PutR and proline levels do not influence *relBE1* expression

**Figure 5:**
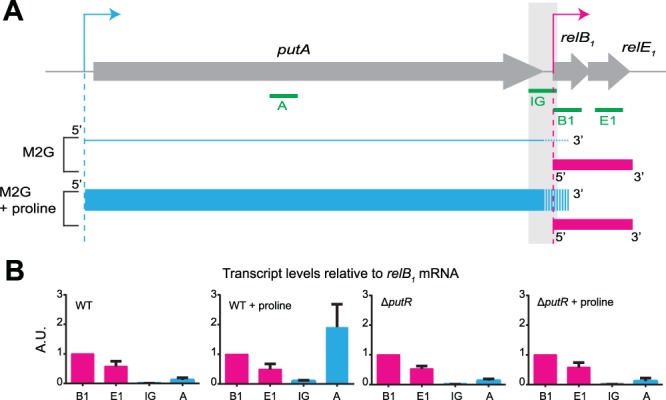
*putA* and *relBE*_1_ are mostly transcribed from independent and differentially regulated promoters. (**A**) Schematic showing the organization of the *putA* chromosomal region including the *relBE*_1_ genes. Grey arrows represent open reading frames. The blue and pink arrows represent the *putA* and the *relB*_1_ promoters, respectively. Green lines show the position of each region (160–200 base pairs) amplified during quantitative RT-PCR (qRT-PCR) assays. The grey shade highlights the IG region amplified by qRT-PCR. Blue and pink lines are a representation of transcripts (mRNA) detected by qRT-PCR and controlled by the *putA* or *relB*_1_ promoters, respectively, when WT cells were cultivated in minimal medium containing 0.2% glucose (M2G) supplemented or not with proline. The weight of blue and pink lines is proportional to the levels of detected transcripts. (**B**) Transcripts from the *putA-relBE*_1_ region detected by qRT-PCR from WT (NA1000) or Δ*putR* (JC1040) cells cultivated in M2G supplemented or not with 10 mM proline. On each graph, the relative abundance of the *relB*_1_ mRNA was arbitrarily set to 1 (A.U.). The other values represent the abundance of specific transcripts relative to the level of *relB*_1_ mRNA in the same strain and growth condition. Error bars represent standard deviations from minimum three independent experiments performed in triplicates.

Interestingly, we noticed that the *relBE*_1_ genes, encoding a functional toxin-antitoxin (TA) module^34^, are located right after the *putA* gene on the *C. crescentus* genome (Fig. 5A). In addition, an RNA-sequencing experiment suggested that *putA* and *relBE*_1_ may belong to the same polycistronic transcript^35,36^. Then, an excess of PutR might result in an excessive transcription of *relBE*_1_, potentially leading to RelE_1_-mediated toxicity on plates or to cell division defects. It is however important to mention that the *relBE*_1_ genes are also transcribed from their own promoter located just upstream of the *relB*_1_ gene (Fig. 5A)^34^. To test if this second promoter is dependent on PutR and/or proline like the *putA* promoter, we constructed a transcriptional fusion between the *relB*_1_ promoter and the *lacZ* gene, and introduced this construct into wild-type and Δ*putR* strains. These strains were then cultivated in M2G medium supplemented or not with proline and promoter activity was evaluated by β-galactosidase assays. We found that the *relB*_1_ promoter was neither dependent on PutR, nor on the presence of proline in the culture medium (Fig. 2E). If *relBE*_1_ and *putA* do belong to the same operon^35^, it was however still possible that *relBE*_1_ transcription might be affected by PutR and proline through their regulation of the *putA* promoter (Fig. 2C,D). To test this possibility, we performed quantitative reverse transcription PCR (qRT-PCR) with four different probes allowing us to distinguish transcripts carrying *putA*, *relB*_1_, *relE*_1_ or the intergenic region between *putA* and *relB*_1_ (IG) independently (Fig. 5A). First, these experiments confirmed that *putA* transcription is strongly dependent on PutR and proline (Fig. 5B), as previously shown using transcriptional reporters (Fig. 2C,D). Second, it showed that the IG region is much less transcribed than the *putA* gene in all tested conditions (Fig. 5B), indicating that transcription starting at the *putA* promoter mostly ends before the *relB*_1_ gene (Fig. 5A). All together, these qRT-PCR results demonstrate that the *relBE*_1_ TA module is only transcribed from the PutR-independent *relB*_1_ promoter under standard growth conditions. Then, it is unlikely that the overproduction of PutR will affect *relBE*_1_ expression, leading to defects in colony formation (Fig. 4) or in cell division (Fig. 3). Supporting this proposal, we observed that the over-production of PutR in a *relBE*_1_ deletion strain still inhibits colony formation on solid media (Fig. 4) and cell division in liquid media (Fig. 3). All together, these results suggest that PutR has other targets than the *putA* promoter and that PutR may not always need proline as an effector.

## Discussion

This study aimed at characterizing proline catabolism and its control in *C. crescentus*. Such a control is critical to ensure that cells can use proline as carbon or nitrogen sources, but that they do not degrade proline when endogenous levels are too limiting. This is all the more important for environmental bacteria like *C. crescentus* that thrive in oligotrophic environments where amino-acids often serve as major carbon sources. Also, the catabolism of proline by PutA enzymes can generate hydrogen peroxide, which can be deleterious to cells^4–7^. We showed that the PutR regulator plays a key role in this process and unexpectedly also discovered that it may affect other PutA-independent cellular functions.

**Figure 6:**
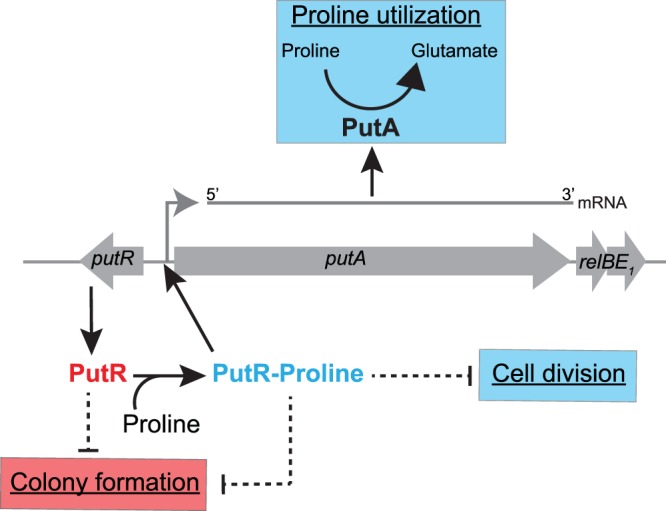
Model for the various roles of PutR in *C. crescentus*. The activation of *putA* transcription by PutR is necessary for the catabolism of proline into glutamate, corresponding to the first step of the proline utilization pathway. In addition, excess of PutR can inhibit cell division and colony formation through still unknown pathways (dashed arrows). PutR requires proline to activate *putA* transcription and inhibit cell division, while proline is dispensable for its effect on colony formation.

The *putA* gene of *C. crescentus* encodes a bi-functional proline dehydrogenase (Figs 1 and 6). Although indispensable for growth on minimal media containing proline as the only carbon source (Fig. 1), we observed that the absence of PutA does not affect growth or cell morphology in complex PYE media where amino-acids serve as major carbon sources (Supplementary Figs S1 and S2). However, *C. crescentus* may frequently find conditions where proline becomes an important source of nutrients in its natural oligotrophic environment. Indeed, *Alphaproteobacteria* have been shown to be often exposed to environments where proline catabolism by PutA becomes indispensable, such as during nodulation or infection by *Sinorhizobium meliloti* and *B. abortus*, respectively^8,9^.

As in many *Alphaproteobacteria*, the *putA* gene of *C. crescentus* is located immediately next to a gene that is divergently transcribed and which encodes a putative DNA binding protein of the Lrp/AsnC family. We found that this gene encodes a strong activator of *putA* transcription in the presence of proline (Figs 2 and 5) and thus named it PutR for proline utilization regulator. Without PutR, the levels of PutA are so low that *C. crescentus* can no more use proline as a carbon source (Fig. 1). The fact that PutR requires proline to activate *putA* transcription (Figs 2 and 5) suggests that proline acts as an effector molecule to stimulate PutR activity. Transcription factors from the AsnC/Lrp family often require such effector molecules to bind to some of their promoter targets, most likely through conformational changes^16^ that may also take place with *C. crescentus* PutR.

Our observation that the *putA* promoter is efficiently activated by proline but still efficiently repressed without proline (Fig. 2C), suggests that this promoter could be used as a useful genetic tool for inducible protein expression in *C. crescentus* cells cultivated in minimal media. Compared to the two other promoters currently available for such applications (from the *xylX* and *vanA* genes)^37^, the *putA* promoter appears to have properties similar to the *xylX* promoter^38^ in terms of efficiency of induction and repression. Moreover, the *putA* promoter does not respond to xylose levels (Fig. 2B), making the combined use of *xylX* and *putA* promoters, to induce the expression of two different proteins, potentially feasible.

Interestingly, several members of the Lrp/AsnC family of transcription factors are so-called global regulators controlling large regulons. The best characterized example is the leucine-responsive protein Lrp of *E. coli* that directly regulates ~130 genes encoding proteins involved in various processes such as metabolism, pili synthesis or adhesion to host cells^39–41^. Other members of this family are more specific regulators, like PutR homologs from a few other *Alphaproteobacteria* such as *A. tumefaciens*, *S. meliloti* and *R. capsulatus*^9,11,14,15^. In *C. crescentus*, we observed that an overproduction of PutR leads to cell division and colony formation defects, which are independent of the activation of *putA* by PutR (Figs 3 and 4). This observation indicates that PutR may have other activities in *C. crescentus*, despite no apparent defects in growth or cell morphology for Δ*putR* cells cultivated with or without proline (Fig. 1 and Supplementary Figs S1 and S2). Future experiments should notably aim at determining under which conditions *C. crescentus* may use this other activity to block or slow down cell division in response to proline levels. Interestingly, recent observations made comparing the phenotypes of Δ*putA* and Δ*putR* strains of *B. abortus* also lead to conclude that PutR may have additional functions beyond simply regulating *putA* transcription, such as adaptation to oxidative stress^9^.

Interestingly, we observed that the inhibition of cell division by PutR was dependent on proline (Fig. 3), while the inhibition of colony formation on solid media was not (Fig. 4). Also, the proline and PutR-dependent inhibition of cell division (Fig. 3) in liquid media is not associated with a drop of cell viability as shown by live/dead staining assays and microscopy (Supplementary Fig. S4). Then, PutR may carry minimum two other functions unrelated with *putA* activation, with only one of these that is dependent on proline (Fig. 6). Another possibility is that the *putA*-independent target(s) of PutR have more impact when *C. crescentus* is cultivated on solid rather than in liquid media. Importantly, we ruled out the possibility that PutR inhibits cell division through a polar effect on the transcription of the downstream *relBE*_1_ genes (Figs 3 and 5) encoding a functional toxin-antitoxin system in *C. crescentus*^34^. Then, how PutR can inhibit cell division in liquid medium or colony formation on plates through direct or indirect effects remains to be clarified (Fig. 6). As a DNA binding protein, it may bind to other promoter regions to regulate the transcription of other genes or, alternatively, have more general effects on chromosome condensation as other members of the Lrp/AsnC family of transcription factors^16,18^. Consistent with this last possibility, results from flow cytometry experiments looking at the DNA content of elongated *C. crescentus* cells over-expressing PutR, suggest that the elongation of DNA replication may be slowed down or frequently arrested in these cells (Supplementary Fig. S5). This may then interfere with cell division through SOS-dependent or SOS-independent mechanisms that tend to block late steps of the cell division process in *C. crescentus*^32,33^. In agreement with this proposal, we found that the FtsK core divisome protein could still localize at mid-cell in elongated cells over-expressing PutR (Supplementary Fig. S3), suggesting that cell constriction, rather than core divisome assembly is inhibited when PutR is over-expressed. Considering that the proteolysis of the CtrA response regulator, which regulates the initiation of DNA replication and the transcription of several cell division proteins in *C. crescentus*^42^, is induced by proline uptake in the intracellular *Alphaproteobacterium Ehrlichia chaffeensis*^43^, we also checked the intracellular levels of CtrA in elongated *C. crescentus* cells over-expressing PutR, but observed no significant difference compared to control cells (Supplementary Fig. S6). Then, although a common point between *C. crescentus* and *E. chaffeensis* is that proline can affect their cell cycles, it appears to do so using different mechanisms in different *Alphaproteobacteria*.

Our findings on PutR are reminiscent of so-called “moonlighting” proteins that are multifunctional proteins that perform multiple autonomous functions without partitioning these functions into different protein domains^44^. Interestingly, moonlighting proteins are the most often involved in the glycolytic pathway or in the TCA cycle. An example found in *Alphaproteobacteria* is the GdhZ moonlighting enzyme that not only converts glutamate into α-ketoglutarate, but also inhibits FtsZ polymerization to modulate cell division in response to glutamate levels^21,45^. Although an excess of PutR in cells exposed to high proline levels may lead to an imbalance in glutamate intracellular levels through PutA, we ruled out the possibility that this is responsible for the observed cell division defect since PutR-overexpressing cells lacking *putA* were still elongated (Fig. 3).

Altogether, one of our surprising finding was that *C. crescentus* PutR may connect the catabolism of proline with other cellular functions such as cell division, but in a PutA-independent manner (Fig. 6). Recent discoveries also highlighted other unexpected connections between cell division and the central metabolism in diverse bacteria^25^. Beyond the example of the GdhZ enzyme^21^, it has, for example, been shown that intracellular α-ketoglutarate levels can influence peptidoglycan synthesis and cell division^22^ or that the transcription of the *ftsZ* cell division gene is influenced by the glutamine-sensitive phosphotransfer system (PTS^Ntr^)^23,24^ in *C. crescentus*. Also, the pyruvate metabolite has been shown to modulate Z-ring assembly for the division of the Gram-positive *Bacillus subtilis* bacterium^46^. Such connections may play an important role in coordinating bacterial growth with cell division, potentially participating to cell size homeostasis^26^.

## Materials and Methods

### Bacterial strains and growth conditions

*C. crescentus* strains used in this study are described in Table 1. The TOP10 *E. coli* strain (Invitrogen, USA) was used for plasmid constructions. *C. crescentus* was cultivated at 28 °C (liquid cultures, with constant shaking) or 30 °C (solid media) in peptone yeast extract (PYE) complex medium or in M2 minimal medium containing 0.2% glucose (M2G), 10 mM proline (M2P) or both (M2GP). For solid media, 1.5% agar (A) was added. *E. coli* was cultivated at 37 °C in LB broth or on LBA. When required, antibiotics where used at the following concentrations (µg.mL^−1^) in liquid media (LM) or solid media (SM) for *C. crescentus*: oxytetracycline (OxyTet):1 in LM and 2 on SM; chloramphenicol (Cam): 1 in LM and on SM; kanamycin (Km): 5 in LM and 25 on SM; spectinomycin (Spec): rich medium = 25 in LM and 100 on SM, minimal medium = 400 in LM and 800 on SM; streptomycin (Strep): rich medium = 5 in LM and on SM, minimal medium = 20 in LM and 40 on SM.

**Table 1:** Strains and plasmids used in this study.

Strain	Genotype	Description	Source or reference
***Caulobacter crescentus***			
CB15	WT’	Wild-type *Caulobacter crescentus* strain	^51^
NA1000	WT	CB15N wild-type strain derivative of CB15	^28^
JC1040	Δ*putR*	NA1000 strain with deletion of *putR*	This study
JC1695	Δ*putA*	NA1000 strain with in frame deletion of *putA*	This study
FC917	Δ*relBE*_1_	CB15 strain with in frame deletion of *relBE*_1_	^34^
LS4200	*xylX*::*ftsK-gfp*	NA1000 strain with *ftsK-gfp* under the control of the native *xylX* promoter	^52^
**Plasmids**			
pNPTS138		Suicide vector containing the *sacB* gene, Km^R^	D. Alley, unpublished
pNPTS138-*putA*		Carrying the regions upstream and downstream of *putA* to create the Δ*putA* strain	This study
pNPTS138-*putR*		Carrying the regions upstream and downstream of *putR*	
pBOR		Vector carrying a Spectinomycin/Streptomycin cassette (Ω), derivative of pHP45Ω	^53^
pNPTS138-*putR*:Ω		Carrying the Ω cassette in between the regions upstream and downstream *putR* to create the Δ*putR* strain	This study
pRXMCS6		Low copy number vector with the *xylX* promoter (P*xyl*), Cam^R^	^37^
pRX-*putR*		*putR* gene under the control of P*xyl* inserted into pRXMCS6 plasmid	This study
pBXMCS6		Medium copy number vector with the *xylX* promoter (P*xyl*), Cam^R^	^31, 37^
pX-*putR*		*putR* gene under the control of P*xyl* inserted into pBXMCS6 plasmid	This study
p*lacZ*290		Low copy number vector carrying the *lacZ* gene, Oxytet^R^	^54^
p*lacZ*290-*putA*P		*lacZ* reporter gene controlled by the *putA* promoter on the p*lacZ*290 plasmid	This study
p*lacZ*290-*relB*_1_P		*lacZ* reporter gene controlled by the *relB*_1_ promoter on the p*lacZ*290 plasmid	This study

### Plasmid constructions

Plasmids used in this study are described in Table 1 and their construction is described below. All PCR amplifications were done using NA1000 genomic DNA and the KOD hot start DNA polymerase (Merck, Germany).

#### Construction of p*lacZ*290-*putA*P and p*lacZ*290-*relB1*P

The promoter regions of *putA* (445 base pairs upstream of the annotated translational start site) or *relB*_1_ (568 base pairs upstream of the annotated translational start site) were amplified using primers described in Supplementary Table S1, carrying *Eco*RI, *Pst*I or *Xba*I restriction sites. PCR products and the p*lacZ*290 vector were digested by *Eco*RI and *Xba*I or *Pst*I. Promoters were then ligated into the vector, giving p*lacZ*290-*putA*P and p*lacZ*290-*relB*_1_P.

#### Construction of pX-*putR* and pRX-*putR*

The *putR* coding sequence (464 base pairs) was amplified using primers described in Supplementary Table S1, carrying *Nde*I, *Eco*RI or *Nhe*I restriction sites. The amplified *putR* sequence and the pBXMCS6 or the pRXMCS6 vectors were digested by *Nde*I *and Nhe*I *or Eco*RI. The *putR* sequences and the vectors were then ligated together, giving pX-*putR* and pRX-*putR*. With these plasmids, *putR* expression is controlled by the *xylX* promoter^31^, which is induced in the presence of xylose in the medium and repressed in the presence of glucose in the medium.

#### Construction of pNPTS138-*putA*, pNPTS138-*putR* and pNPTS138-*putR*:Ω

From 500 to 730 base pairs upstream (UP) and downstream (DOWN) of the *putA* and *putR* coding sequences were amplified using primers described in Supplementary Table S1. PCR products were digested by *Eco*RI, *Bam*HI and *Nhe*I (*putA*) or *Pst*I, *Bam*HI and *Nhe*I (*putR*). Digested UP and DOWN sequences were then both ligated into a pNPTS138 vector digested with the same enzymes, giving pNPTS138-*putA* and pNPTS138-*putR*. An Ω cassette extracted from pBOR digested by *BamHI* and encoding Spec/Strep resistances was then introduced at the *Bam*HI restriction site between the UP and the DOWN sequences of *putR*, giving pNPTS138*-putR*:Ω.

### Strain constructions

Plasmids were introduced into NA1000 or CB15 *C. crescentus* strains as published in^47^.

To construct the Δ*putA* deletion strain, the pNPTS138-*putA* plasmid was integrated into the *C. crescentus* chromosome at the *putA* locus by single homologous recombination, selecting for Km-resistant colonies. The resulting strain was grown to stationary phase in PYE medium lacking Km. Cells were plated on PYEA + sucrose 3% and incubated at 28 °C or 30 °C. Single colonies were picked and transferred in parallel onto PYEA plates containing or not Km. Km-sensitive clones, which had lost the integrated plasmid due to a second recombination event were isolated. Colonies were further tested for their capacity to grow on M2GA medium but not on M2PA medium.The Δ*putA* deletion was then verified by colony-PCR using specific primers.

To construct the Δ*putR* deletion strain, the pNPTS138-*putR:Ω* plasmid was integrated at the *putR* locus of the chromosome of a *C. crescentus* NA1000 strain by single homologous recombination, selecting for Km-resistant colonies. The pRX-*putR* plasmid was then introduced into this strain. The resulting strain was grown to stationary phase in PYE medium supplemented or not with 0.3% xylose and Cam, but lacking Km. Cells were plated on PYEA + sucrose 3% + xylose 0.3% or 0.1% + Cm and incubated at 28 °C. Single colonies were picked and transferred in parallel onto PYEA + xylose 0.3% + Cam + Strep + Spec or onto PYEA + xylose 0.3% + Cam + Km. Km-sensitive and Spec/Strep-resistant clones, which had lost the integrated plasmid due to a second recombination event were isolated. These were supposedly Δ*putR*::Ω. The Δ*putR*::Ω deletion was then verified by colony-PCR using specific primers and transduced into a NA1000 WT strain using ΦCR30 phages^48^ selecting for Strep/Spec-resistances, giving the so-called Δ*putR* strain.

### β-galactosidase assays

β-galactosidase assays were done using standard procedures as previously described^49^.

### RNA extraction

Total RNA were extracted from cultures at an OD_660_ of 0.6, using the RNeasy miniprep kit (Qiagen, GER) including the recommended DNase I treatment. A second DNase I treatment was done to remove the remaining gDNA using the Turbo DNase I (Ambion, USA) at 37 °C for 30 minutes and RNA were then purified using the RNeasy MinElute Kit (Qiagen, GER). RNA samples were tested for purity by PCR. RNA quantity was measured using a Nanodrop fluorospectrometer, while quality was checked by agarose gel electrophoresis.

### Quantitative Real-Time PCR (qRT-PCR) analysis

cDNA were synthetized from 5 µg of total RNA using the Superscript III reverse transcriptase and 250 ng of random hexamers according to the manufacturer’s instructions (Invitrogen, USA). The qRT-PCR was performed using the GoTaq qPCR master mix (Promega, USA), 2 μl of cDNA (diluted 1:100) and 0.5 µM of specific primers (Supplementary Table S1) for each gene. The enzyme was activated by heating at 95 °C for 2 minutes. Then, the cDNA was amplified by qPCR (40 cycles) with a denaturation step at 95 °C for 15 seconds, an annealing step at 60 °C for 60 seconds. A final step of dissociation was achieved by increasing the temperature by 0.3 °C every 5 seconds from 60 °C to 95 °C. qPCRs were performed in triplicates using a Rotor-GeneQ instrument (Qiagen, GER). Melting curves were analyzed and the presence of a single peak was checked. For each pair of primers, primer efficiency was evaluated using standard curves from gDNA from 5 ng/µL to 0.001 ng/µL. Absolute quantifications x were determined using the following formula: x = (y − b)/m; y corresponds to the Ct value, while m and b are automatically calculated using standard curves.

### Microscopy

Cells were immobilized on slides using a thin layer of PYE + 1.5% agar and imaged immediately. Phase contrast microscopy images were taken with a Plan-Apochromat 100X/1.45 oil Ph3 objective on an AxioImager M1 microscope (Zeiss) with a Prime-95B back-illuminated CMOS camera (Photometrics) controlled by the VisiView software (Visitron Systems, Germany). Images were processed using Adobe Photoshop. The MicrobeJ software^50^ was used to measure the length of cells on images using default parameters. Measurements from 100–200 cells were used to evaluate cell size distributions.

## Electronic supplementary material

Supplementary information
