# Supplementary material for: Control of proline utilization by the Lrp-like regulator PutR in Caulobacter crescentus
Source: Sci Rep. 2018 Oct 2;8:14677. doi: 10.1038/s41598-018-32660-3 (PMC6168545; doi:10.1038/s41598-018-32660-3)
Supplement: Supplementary file 1 — Supplementary information [file 41598_2018_32660_MOESM1_ESM.pdf]

Control of proline utilization
by the Lrp-like regulator PutR in *Caulobacter crescentus*

Annabelle Mouammine, Katharina Eich, Antonio Frandi and Justine Collier

Supplementary material

Supplementary Figures:

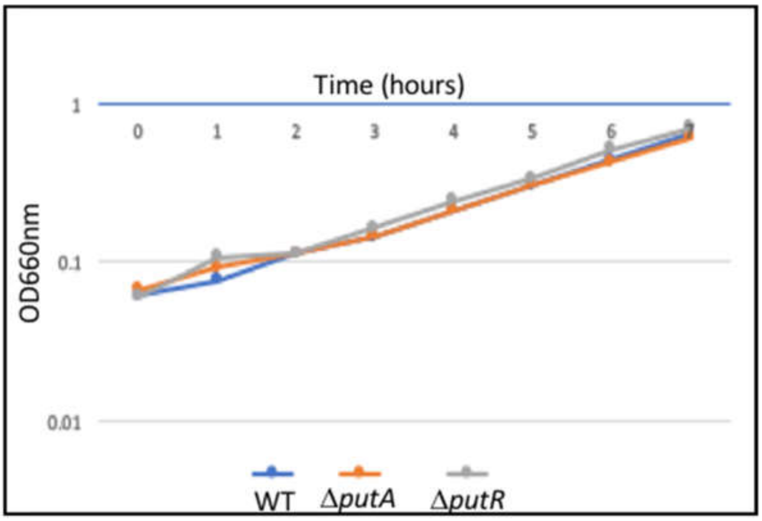

Figure S1: Δ*putA* and Δ*putR* cells grow at a rate similar to WT cells in complex medium. Growth of WT (NA1000), Δ*putA* (JC1695) and Δ*putR* (JC1040) strains in exponential phase in liquid PYE medium. Each curve represents the mean of three independent growth curves.

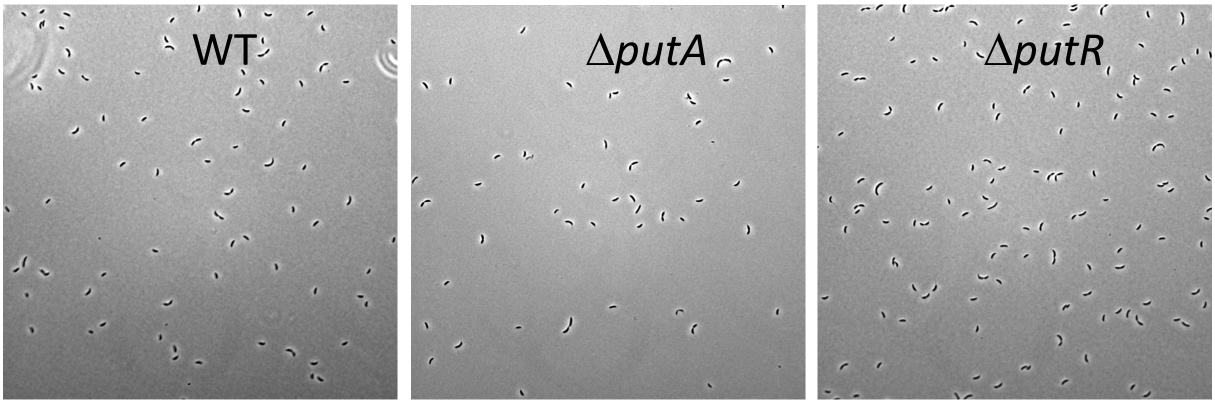

Figure S2: Δ*putA* and Δ*putR* cells do not display apparent morphology defects when cultivated in complex medium. WT (NA1000), Δ*putA* (JC1695) and Δ*putR* (JC1040) cells were cultivated in exponential phase in liquid PYE medium (Fig.S1). Phase contrast images were taken when cells reached an OD660nm of ~0.3 as described in Material and Methods.

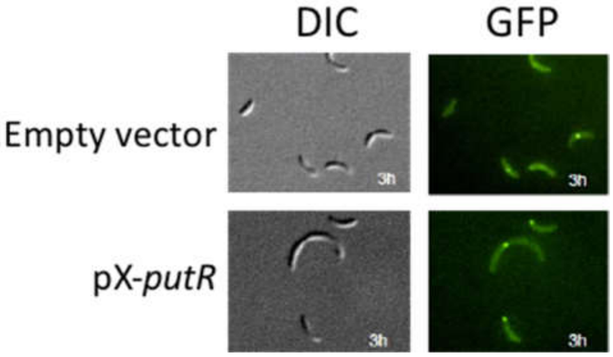

Figure S3: Excess of PutR does not prevent the localization of FtsK-GFP to mid-cell in complex medium. Cells from strain LS4200 (NA1000 *xylX*::*ftsK-gfp*) carrying pBXMCS6 (empty vector) or pX-*putR* were cultivated in PYE + glucose 0.2%. 0.3% of xylose was added to cells in exponential phase and cells were incubated for 3 hours before imaging as previously described [1].

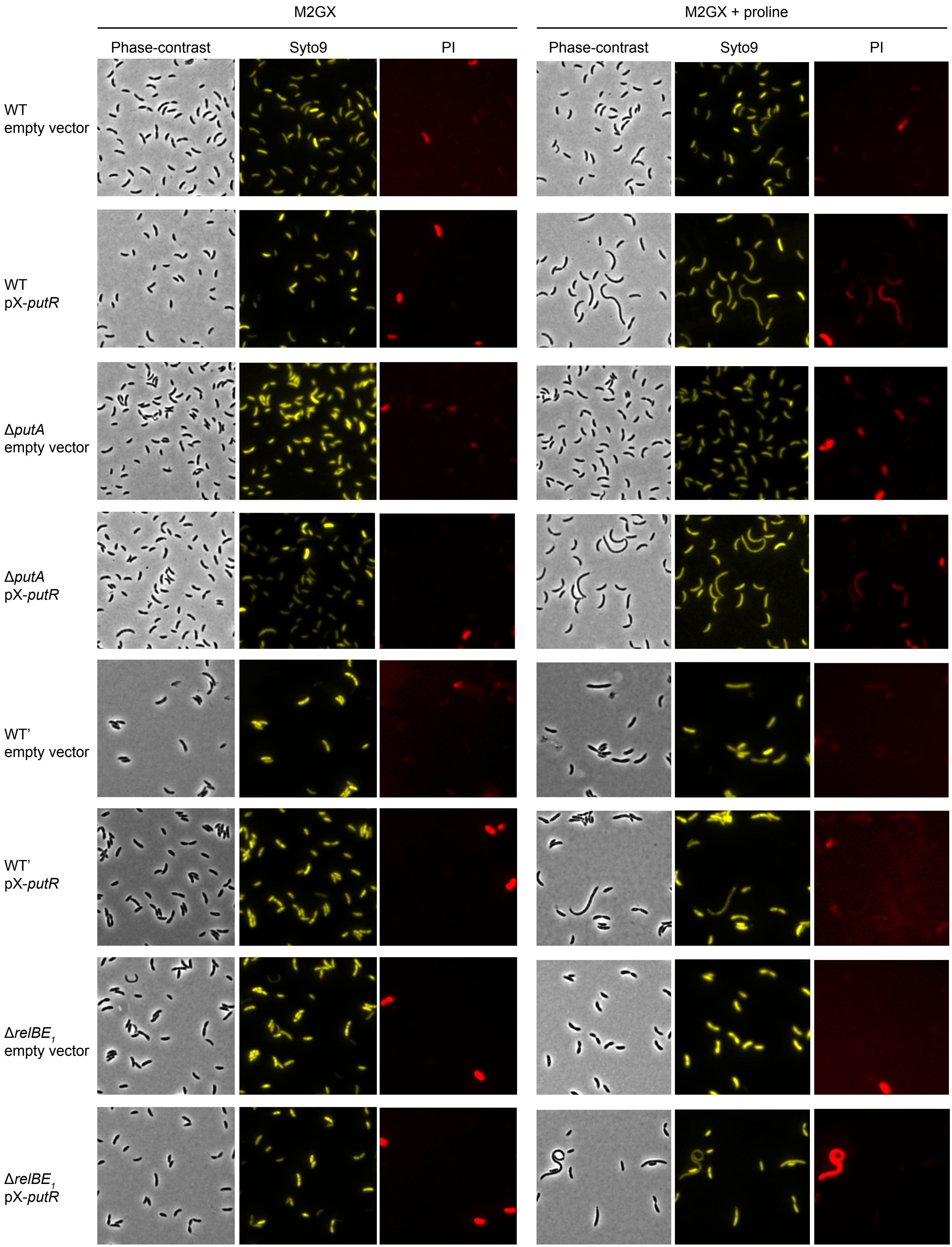
M2GX
M2GX + proline
Phase-contrast
Syto9
PI
Phase-contrast
Syto9
PI
WT
empty vector
WT
pX-putR
ΔputA
empty vector
ΔputA
pX-putR
WT'
empty vector
WT'
pX-putR
ΔrelBE1
empty vector
ΔrelBE1
pX-putR

Figure S4: Cell division defects induced by excess PutR do not affect cell viability. NA1000 (WT) and isogenic Δ*putA* cells, or CB15 (WT') and isogenic Δ*relBE₁* cells, carrying the pBXMCS6 (empty vector) or the pX-*putR* plasmid were cultivated in M2G and diluted the next morning into M2G supplemented or not with proline. Once cultures reached an OD_{660nm} of ~0.25, 0.3% xylose (M2GX) was added to induce the expression of *putR* from pX-*putR* over-night. Cells were then stained with Syto9 and Propidium iodure (PI) and imaged by phase-contrast and fluorescent microscopy. All cells get stained with Syto9 while only dead cells get stained with PI. Representative images from three independent experiments are shown in this figure.

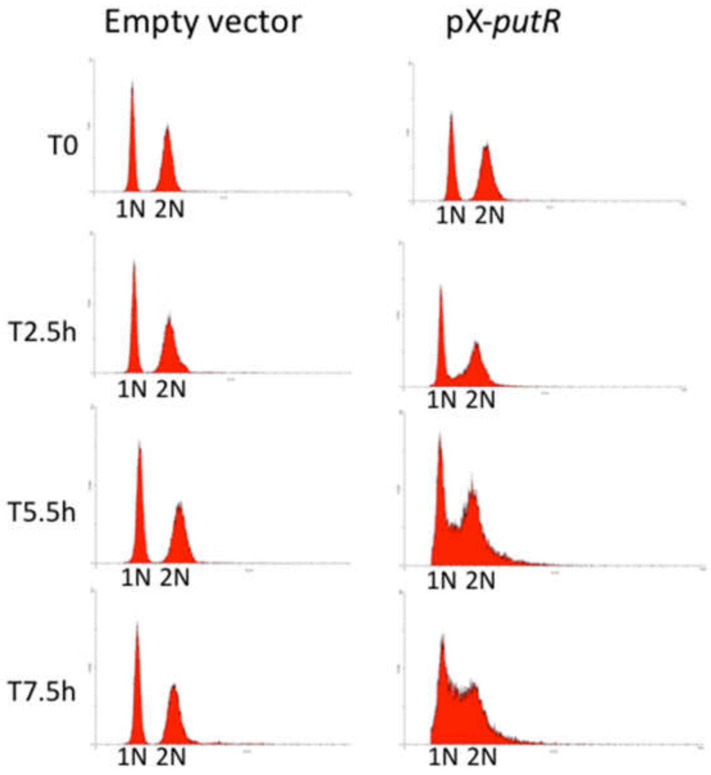

Figure S5: Excess of PutR may lead to replication elongation defects in cells cultivated in complex medium. NA1000 cells carrying pBXMCS6 (empty vector) or pX-*putR* were cultivated in PYE + glucose 0.2%. 0.3% of xylose was added to cells cultivated in exponential phase and cells were incubated for the indicated time period. Rifampicin (15µg/mL) was then added to cultures and incubated for four hours (T0 and T2.5h) or over-night (T5.5h and T7.5h) to block news rounds of replication. Samples were then fixed with 80% ethanol. DNA staining and flow cytometry analysis were then performed as described before [2]. The horizontal axis indicates the number N of complete chromosomes: 1N or 2N. The vertical axis indicates the number of cells. The same scale was used for each graph. The presence of incompletely replicated chromosomes (1N<DNA content<2N) hours after rifampicin addition suggests that the elongation of DNA replication was slowed down or frequently arrested in cells over-expressing PutR in complex medium.

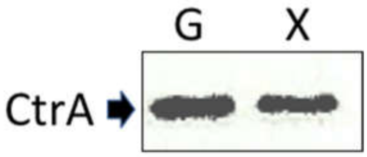

Figure S6: Excess of PutR does not significantly influence intracellular levels of CtrA in cells cultivated in complex medium. Immunoblot analysis comparing the intracellular levels of CtrA in NA1000 cells carrying the pX-*putR* plasmid. Cells were cultivated over-night in PYE + 0.2% glucose and the culture was diluted into fresh PYE + 0.2% glucose (G) to an OD660nm of ~0.05. 0.3% of xylose was then added into half of the culture (X) and both halves were incubated for 5 hours before sampling at an OD660nm of ~0.3. As expected, cells cultivated in the presence of xylose showed cell division defects.

Supplementary Material and Methods:

Live-dead microscopy

Live-dead staining procedures were performed using the LIVE/DEAD *Bac*Light Bacterial Viability Kit (Thermofisher-scientific, USA) following the manufacturer's instructions. Stained cells were incubated in the dark for 5 minutes before imaging as described in the main text. A YFP filter was used to detect Syto9 and a RFP filter was used to detect PI. Images were processed using Adobe Photoshop and Image J.

Immunoblot analysis

The CtrA protein was resolved on 12% SDS/PAGE [3]. Gels were electrotransfered to a PVDF membrane (Millipore). Immunodetection was performed with polyclonal antobodies. Anti-CtrA and anti-rabbit conjugated to horse-radish peroxidase (Sigma Aldrich) sera were diluted 1:20'000. Chemiluminescence detection was done as described previously [2].

Supplementary Table:

Table S1: List of primers used in this study

Primer name	Primer sequence (5' to 3')	Use
L-*putA*P	ATCGAGTTTTCACCGAAGAAGCTT	Construction of p*lacZ*290-*putA*P
R-*putA*P	TATACCAACAAGCGGAAACGACC	
L- $relB_1$P	AAAAAAGAATTCGACAAGCATCTGGTTCGCC	Construction of p*lacZ*290-$relB_1$P
R-$relB_1$P	AAAAAATCTAGAGCAACGACCTTCAGCC	
L-Δ*putR*-UP	AACTGCAGGCTTGCGGACGCTGCGGCGGG	Construction of pNPTS138-*putR*::Ω plasmid
R-Δ*putR*-UP	CGGGATCCGTTTTCACCGAAGAAGCTTCGAAA	

L-Δ*putR*-Down	CGGGATCCCGTCACCGCTCAGATCAGGCCT	
R-Δ*putR*-Down	CTAGCTAGCGTGGCGACCTATGAGAAGACGG	
L-*putR*-low	GGAATTCCATATGGCCATTTCTGCTCTAGATGA	Construction of pRX-*putR* plasmid
R-*putR*-low	CTAGCTAGCTAGAGCGGCAGTTGCACCGT	
L-*putR*-Med	GGAATTCCATATGGCCATTTCTGCTCTAGATGA	Construction of pX-*putR* plasmid
R-*putR*-Med	GGCGAATTCTAGAGCGGCAGTTGCACCGT	
L-Δ*putA*-UP	GCGGATCCGCCGTCGGACTGCAGAACCTTC	Construction of pNPTS-*putA* plasmid
R-Δ*putA*-UP	GCGAATTCGGTCGTTTCCGCTTGTTGGTATA	
L-Δ*putA*-Down	GCGAATTCCCCTTGACGCCATCGGCCATCG	
R-Δ*putA*-Down	GCGCGCTAGCGGATCACATAGGCGCTCTGACC	
L-B1	TATGTCCGTGTCCGAGTACG	qRT-PCR of *relB1* from NA1000 (161 bp)
R-B1	TATGAGCGCGGAACTCATCG	
L-E1	TTCAACCGTCTCATCGTCTG	qRT-PCR of *relE1* from NA1000 (193 bp)
R-E1	ATCACATAGGCGCTCTGACC	
L-IG	GCTCAGCGTCAACATCACC	qRT-PCR of *putA-relB1* IG region from NA1000 (187 bp)
R-IG	CGTACTCGGACACGGACATA	
L-A	CGGATCTGTCGTACCTCGTC	qRT-PCR of *putA* from NA1000 (185 bp)
R-A	GTCGGCGGCCTTATAGAGAG	

Supplementary References:

1 Fernandez-Fernandez, C., Grosse, K., Sourjik, V. & Collier, J. The beta-sliding clamp directs the localization of HdaA to the replisome in *Caulobacter crescentus*. *Microbiology* **159**, 2237-2248, doi:10.1099/mic.0.068577-0 (2013).

2 Fernandez-Fernandez, C., Gonzalez, D. & Collier, J. Regulation of the Activity of the Dual-Function DnaA Protein in *Caulobacter crescentus*. *PLoS One* **6**, e26028, doi:10.1371/journal.pone.0026028
PONE-D-11-13802 [pii] (2011).
3 Sambrook, J., Fritsch, E.F., and Maniatis, T. *Molecular Cloning: a Laboratory Manual*. (Cold Spring Harbor Laboratory Press, 1989).
